# Mini‐Linguistic State Examination (MLSE): Preliminary Normative Data for the French‐Canadian Version of an International Screening Tool for Primary Progressive Aphasia

**DOI:** 10.1002/alz70857_101028

**Published:** 2025-12-25

**Authors:** Elizabeth Poulin, Monica Lavoie, Lorraine Bavelier, Sophie Ferrieux, Lucie Grimont, Isaure De Marcellus, Cécile Rebillard, Marc Teichmann, Robert Laforce

**Affiliations:** ^1^ Research Chair on Primary Progressive Aphasia ‐ Fondation de la famille Lemaire, Quebec, QC, Canada; ^2^ Clinique Interdisciplinaire de mémoire, CHU de Québec ‐ Université Laval, Quebec City, QC, Canada; ^3^ National Reference Center for « Rare or Early Onset Dementias », Pitié‐Salpêtrière University Hospital, Assistance Publique Hôpitaux de Paris (AP‐HP), Paris, France

## Abstract

**Background:**

There is a need for internationally applicable screening tests assessing language in neurodegenerative conditions, allowing for homogenized diagnosis/classification of primary progressive aphasias (PPA), monitoring language decline, and providing endpoints in clinical/therapy trials. The ‘Mini‐Linguistic State Examination’ (MLSE) is currently developed within a worldwide network including 22 countries, and our Paris‐Québec collaboration aimed at developing the French/Canadian MLSE (fc‐MLSE), validating it with healthy controls and clinical populations.

**Method:**

The fc‐MLSE was adapted from the English version using stimuli of similar linguistic complexity. As the English version, the fc‐MLSE included 11 sub‐tests assessing 5 linguistic domains (motor‐speech, phonology, semantics, syntax, verbal working‐memory), providing a total score (/100) and 5 sub‐scores. The test was administered to 182 healthy controls to generate normative scores, and subsequently to 36 patients with PPA [(nonfluent/agrammatic variant (nfvPPA, *n* = 8), logopenic variant (lvPPA, *n* = 20), semantic PPA (svPPA, *n* = 8)], and 6 patients with Alzheimer's disease (AD).

**Result:**

Testing durations were approximately 8 and 12 minutes for controls and patients, respectively, and the inter‐rater consistency was of 92%. In controls, no ceiling effects were observed. The total score and the 5 sub‐scores were similar for women/men, and led to stratifications according to age‐ranges and educational levels. In clinical populations, the fc‐MLSE was able to distinguish AD and PPA patients from controls, and PPA had lower total MLSE scores than AD patients. Sub‐scores also distinguished the three PPA variants, showing highest error‐rates for motor speech in nfvPPA, verbal working‐memory in lvPPA, and semantics in svPPA.

**Conclusion:**

The fc‐MLSE is a rapid and examiner‐consistent language test, validated with a large population of healthy controls. It can be helpful for clinical classification of PPA variants, and might represent a valuable tool for follow‐up trial monitoring in PPA, AD and other neurodegenerative diseases affecting language. More generally, the use of MLSE versions developed in 22 countries will improve the consistency/uniformity of language assessments at the international level.

